# Automated Spatial Pattern Analysis for Identification of Foot Arch Height From 2D Foot Prints

**DOI:** 10.3389/fphys.2018.01216

**Published:** 2018-09-03

**Authors:** Julien Lucas, Kinda Khalaf, James Charles, Jorge J. G. Leandro, Herbert F. Jelinek

**Affiliations:** ^1^Department of Biology and Computer Science, University of Poitiers, Poitiers, France; ^2^Department of Biomedical Engineering, Khalifa University of Science and Technology, Abu Dhabi, United Arab Emirates; ^3^Institute of Koorie Education, Deakin University, Waurn Ponds, VIC, Australia; ^4^School of Medicine, Deakin University, Waurn Ponds, VIC, Australia; ^5^Institute of Mathematics and Statistics, University of São Paulo, São Paulo, Brazil; ^6^School of Community Health, Charles Sturt University, Albury, NSW, Australia

**Keywords:** non-linear dynamics, complexity, wavelet analysis, bending energy, foot arch height

## Abstract

Arch height is an important determinant for the risk of foot pathology, especially in an aging population. Current methods for analyzing footprints require substantial manual processing time. The current research investigated automated determination of foot type based on features derived from the Gabor wavelet utilizing digitized footprints to allow timely assessment of foot type and focused intervention. Two hundred and eighty footprints were collected, and area, perimeter, curvature, circularity, 2nd wavelet moment, mean bending energy (MBE), and entropy were determined using in house developed MATLAB codes. The results were compared to the gold standard using Spearman’s Correlation coefficient and multiple linear regression models with significance set at 0.05. The proposed approach found MBE combined with foot perimeter to give the best results as shown by ANOVA (*F*_(2,211)_ = 10.18, *p* < 0.0001) with the mean ±*SD* of low, normal, and high arch being, respectively, 0.26 ± 0.025,.24 ± 0.021, and 0.23 ± 0.024. A clinical review of the new cut off values, as set by the first and the third quartiles of our sample, lead to reliability up to 87%. Our results suggest that automated wavelet-based foot type classification of 2D binary images of the plantar surface of the foot is comparable to current state-of-the-art methods providing a cost and time effective tool suitable for clinical diagnostics.

## Introduction

**FIGURE 1:**
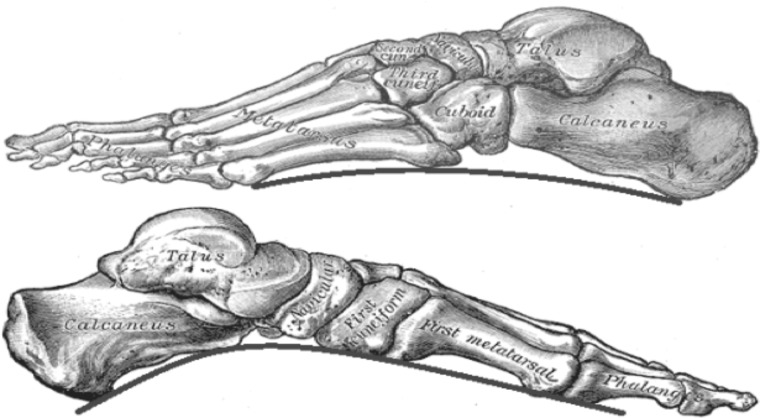
Skeletal structure of the foot. From top to bottom: outline of the lateral arch and medial arch. Adapted from Gray and Lewis (1918).

The arch height of the foot has long been recognized as a key parameter in foot type classification, and is considered an important prediction and diagnostic tool in lower limb pathology. Some studies have shown that high and low arched foot types can alter plantar pressures as compared to a foot with normal arch height (Van Schie and Boulton, 2000). High arched foot type has also been found to be associated with increased levels of foot discomfort and pain (Burns et al., 2005). Due to differences in repeatability and reliability, as well as ease of use, no single method has been fully accepted for objective foot arch assessment. Various invasive, time consuming, and costly measures exist and have been tested as clinical tools to determine arch height (Hawes et al., 1992), but all require either extensive manual pre-processing or post-processing of the images, before results can be obtained. Several non-invasive methods based on footprint images have also been proposed as useful measures in gait and movement analysis (Johnston, 2014). However, these methods require extensive processing, are very time consuming and largely underutilized by clinicians and researchers. In the current paper, we propose an automated method based on Gabor wavelet results of 2- dimensional footprints and compare these to the current gold standard assessment methodology. Wavelet analysis simplifies the determination of the arch index for clinicians by requiring only the ink foot print without toes to be uploaded to the script. The Gabor-wavelet analysis is a common tool used in 2-dimensional pattern analysis as it allows extraction of a number of features that describe a pattern. In the current research we have determined the circularity, histogram of orientation, and mean bending energy (Costa and Cesar, 2001 #623). These features describe the characteristics of the footprint. For example the circularity feature measures the degree of departure from a circle and is therefore sensitive to the medial part of the footprint, which has a straighter appearance in flatfoot and is more rounded closer to a circle with normal arch height. Arch height is a function of several factors, including a complex foot structure of 26 bones, 16 joints, and more than 100 muscles, tendons, and ligaments. The structure and function of the lower limb ensures support and stability in gait, as well as good posture for balance and movement. The skeletal, musculature, and ligamentous components of the foot lead to the formation of the three plantar arches (Goonetilleke, 2012 #15208), which provide flexibility and weight bearing support (**Figure 1**) (Manley and Solomon, 1979, Parker et al., 2015, Gwani et al., 2017). The foot arch of most importance to function and gait is the medial longitudinal arch (MLA), as this arch provides most of the elasticity and stability during gait. The focus of this paper is the development of new innovative, less time consuming method to calculate the height of this arch to assess pathology.

**FIGURE 2:**
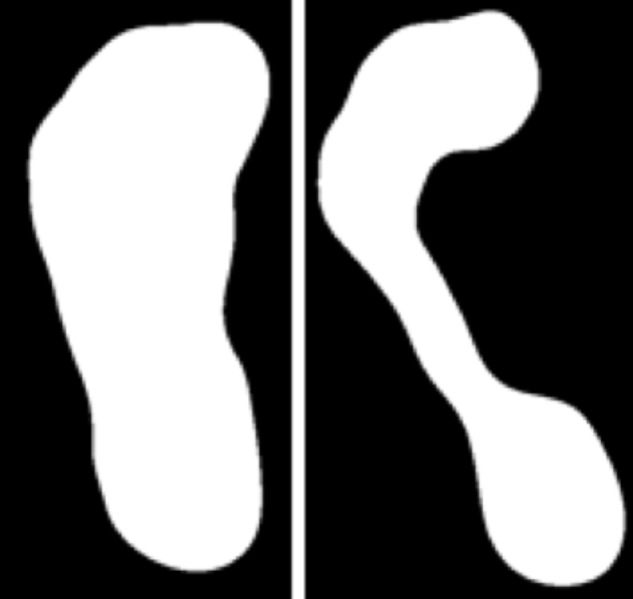
Binary footprint of a flat foot and high arch.

Medial longitudinal arch height is also important in shock absorption, providing support while walking (Ghasemi et al., 2016). Abnormal arch height such as high arches, flat feet or fallen arches (**Figure 2**) can be responsible for discomfort and more serious pathology, such as, lumbar lordosis, foot eversion, and knees injuries (Nigg et al., 1993), plantar fasciitis, tibialis posterior tendon dysfunction (Johnson and Strom, 1989; Schepsis et al., 1991).

Different methods to evaluate arch height exist and have been verified in several studies. The current gold standard method adopted in clinical practice is the “Arch Index” proposed by Cavanagh and Rodgers (1987). The accuracy of this method has been verified by experts in examining arch height (Menz and Munteanu, 2006). However, the C&R method is difficult to use and time consuming. Clarke’s Arch Angle (Clarke, 1933), Index of Irwin (1937), Truncated Arch Index and the Arch Length Index (Hawes et al., 1992) have been proposed as alternatives but have similar shortcomings and are therefore not used routinely in clinical practice. Automated feature analysis methods with Gabor Wavelet-based feature extraction have been applied in diverse clinical areas and have shown promise in identification of plagiocephaly, proliferative retinopathy, brain tumor detection, and complex image analysis (Jelinek et al., 2003; Jelinek et al., 2014; Nagtode et al., 2016). Therefore, the current paper explores the use of automated images analysis for the classification of footprints within a clinical environment and for research purposes. There is a need to develop a computer-based method to establish arch height which is less time consuming and likely to be used by clinicians and researchers. The new innovative approach developed by the authors is an automated, self-contained computer-based analysis method using digitized 2D footprints. The methodology is based on features derived from geometrical characteristics and the use of the Gabor wavelet.

## Materials and Methods

**FIGURE 3:**
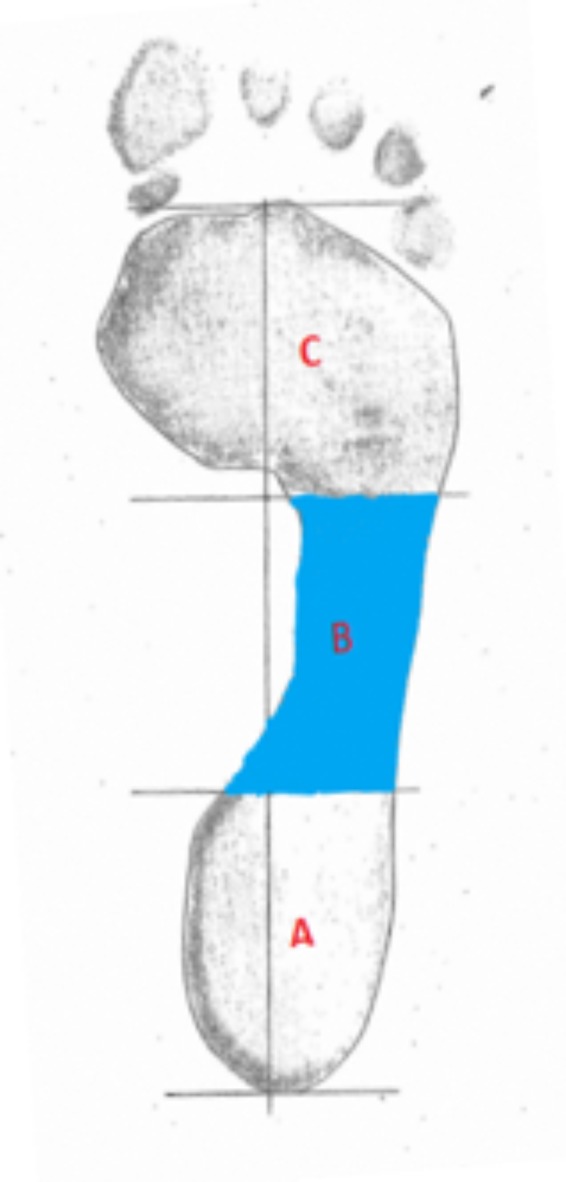
Determination of Arch Index according to Cavanagh and Rogers’ method.

Footprints for analysis were collected from 143 volunteers as part of a foot health screening. MLA height index was determined using a pedograph footprint system (Welton, 1992). A sample of two hundred and eighty ink footprints were collected from the volunteers upon informed and written signed consent. The foot ink prints were taken with a standard Ruckgaber Orthopadie ink plate developed by Ruckgaber Bruggemann^1^. Participants volunteered from three different regions of Australia and were included if they could walk unassisted and have no lower limb pathology, footprints were collected with a standard pedograph of the left and right foot. Ethics approval was granted from the Human Research Ethics Committee (HREC) at the University of Newcastle (Protocol Number 2012–0385) and all participants provided written consent following an information session Initially, the arch index proposed by C&R, representing the current gold standard method, was determined. To obtain the Arch Index (AI) requires a line to be drawn from the middle of the heel to the center of the second toe of the foot. Then the toeless footprint is divided into three equal parts by dividing the longitudinal line into three equal parts through drawing two lines perpendicular to the central longitudinal line (**Figure 3**). The footprint image is then imported into the Analyzing Digital Image analyzing program (ADI, University of Massachusetts Amherst, MA, United States) and the outline of the footprint is traced.

The AI is determined by calculating the area of the middle segment (area of section B) and this is divided by the area of the whole foot (area of section A, B, and C), i.e., B/(A + B + C). A low arch is indicated by an AI being equal to or higher than 0.26, whereas a high arch has an AI of equal or lower than 0.21 (Cavanagh and Rodgers, 1987). To calculate the proposed new features, the outline of the footprints was manually traced and then scanned into the computer for further analysis. An Image Editor tool (IrfanView) was used to remove the toes from the image. All prints were analyzed using an in-house algorithm written in MATLAB (MathWorks, MathWorks Inc., New York, NY, United States). Binary images were obtained by applying image thresholding and Sobel operator to the gray scale image. Then the binary images were uploaded as a batch file to the Feature Analysis Algorithm, to determine perimeter, area, curvature, circularity, 2nd moment, entropy, and bending energy (Van Vliet and Verbeeck, 1993; Costa and Cesar, 2001). Area, perimeter, and circularity features were extracted based on the geometry of the footprint. The wavelet based features, including second moment of the magnitude of the wavelet transform (Arnéodo et al., 2000), entropy-based features of the histogram such as orientation of the wavelet transform and curvature, were consequently determined. Finally, mean bending energy (MBE) and circularity associated with the contour of the footprint were calculated. Using binary images, the perimeter corresponds to the edge between white and black pixels on the image of the foot. The perimeter was calculated by applying an edge detection algorithm, where it was determined by counting the boundary pixels multiplied by π/4. As for area, each line, and column of the digital footprint corresponded to the sum of foreground pixels. The circularity highlights the relationship between perimeter and area, where *P* equals perimeter and A corresponds to the area. The circularity is then defined as:

(1) $$C=\frac{p^{2}}{a}$$

The entropy represents the histogram of the Orientation (angles) of the Wavelet Transform and is a statistical measure of the degree of orientation disorder as follows:

(2) $$E=-\sum_{n=1}^{\infty }p_{i}\text{ In }(p_{i})$$

where p_i_ is the frequency of vectors oriented toward a specific direction, and *k* corresponds to each bin in the histogram. The curvature represents how the direction of a tangent vector varies from point to point on the shape. This feature is given by the following equation:

(3) $$k=▽\cdot \frac{▽f}{\left||▽f|\right|}=\frac{f_{xx}f_{y}^{2}-2f_{x}f_{y}f_{xy}+f_{yy}f_{x}^{2}}{{\left(f_{x}^{2}+f_{y}^{2}\right)}^{3/2}}$$

where *f_x_, f_y_, f_xx_, f_yy,_ and f_xy_* denote the first and the second partial derivatives of *f* with respect to *x* and *y*, and the partial derivatives with respect to *x* and *y*, respectively. Mean Bending Energy (MBE), also known as boundary energy, is related to the amount of energy necessary to transform the shape of the image into a circle, which would have the same perimeter. This feature is a curvature-based shape descriptor, whose discretised version is defined as:

(4) $$M\overset{⌢}{B}E=\frac{1}{N}\sum_{N=0}^{N-1}k{\left(n\right)}^{2}$$

where *N* is the number of pixels in the contour and *k(n)* is the local curvature for the *n*th pixel in the contour. The Gabor wavelet is defined as:

(5) $$\psi_{G}(x)=\exp\;(jk_{0}x)\exp\left(1\frac{1}{2}|Ax{|}^{2}\right)$$

Where $j=\sqrt{-1}$, k_0_ is a vector, which defines the frequency of the complex exponential, and $A=\text{diag}\left[\varepsilon^{-1/2},1\right],\varepsilon \geq$ 1is a 2 × 2 diagonal matrix that defines the anisotropy of the filter, and its elongation in any direction. The Gabor wavelet is a complex exponential modulated by a Gaussian. The above equations were based on the work reported in Costa and Cesar (2001).

Statistical analyses included a correlation analysis using Spearman’s correlation coefficient to determine any collinearity between the proposed features and the C&R Arch Index currently used in clinical practice. Simple and multiple linear regression models were also applied to investigate the relationship between the predictor variables. The relative quality of each statistical model consisted of combinations of the proposed features including perimeter, area, curvature, circularity, 2nd moment, entropy, and bending energy (Van Vliet and Verbeeck, 1993; Costa and Cesar, 2001). For example, the corrected Akaike Information Content (AICc) measures the relative quality of the statistical models for the data, with the smallest AICc indicating the best model. The Variance Inflation Factor was applied to ensure that there was no multicollinearity between factors (Zuur et al., 2010). The correlation coefficient (*r*^2^) was used to prove the reliability of the *AHI* linear equation obtained and shown in the results. The model with the lowest AICc and *p*-value < 0.05 with the least number of features was selected as the best model to describe low, normal and high arch heights and best matched the three groups of C&R (low, normal, and high arches). An Analysis Of Variance (ANOVA) followed by the Tukey HSD *post hoc* (Tukey et al., 1984) test were applied to determine which pairwise groups of models were significantly different. All statistical analyses were carried out in R Studio with significance set at *p* < 0.05.

## Results

Spearman’s correlation test analysis revealed that several of the proposed features obtained using the Gabor wavelet-based analysis were correlated (*r*^2^ > 0.7), including the area and perimeter with circularity, and the second moment of the Magnitude of the Wavelet transform with entropy and curvature (**Table 1**). Of the correlated pair of features, the feature that had a higher correlation with the dependent variable (arch height) was retained for the regression analysis.

**Table 1:** Significant result of spearman’s correlation for the proposed features.

		Area	Perimeter	Second moment
Circularity	Correlation coefficient	**-0.892^∗^**	**0.692^∗^**	
Entropy		0.354	0.014	**-0.683^∗^**
Curvature		**-**0.296	0.089	**0.851^∗^**

*^∗^Correlation is significant at the 0.01 level.*

From all features, the multiple linear regression models were tested in accordance with the statistical models. Every combination of any model was studied. To avoid multicollinearity issues, any model with a Variance Inflation Factor greater than two was eliminated. Results of this regression tests are shown in the following table. Each one of the four models in **Table 2** is equally valid based on the statistical analyses to model the C&R classification. All models had an R-squared value of 0.28 and were significant (*p* < .001).

**Table 2:** Best model regarding cavanagh & rodger classification.

Model	AICc^∗^
MBE + P	-761.68
MBE + E + P	-760.44
MBE + C + P	-760.31
MBE + SM + P	-759.88

*^∗^AICc – corrected akaike information content, MBE – Mean bending energy, P – Perimeter, E – Entropy, C: Circularity, SM: Second moment.*

The optimal model based on the multiple regression analysis, consisted of the combination of Bending Energy (MBE), and Perimeter (P), with the lowest number of features in the equation. The related equation of this model is the following:

(6) $$AHI=(-7.351^{-05}\times P)-\left(1050.964\times M\overset{⌢}{B}E\right)+.4597.$$

The ANOVA was further applied to determine any relationship between our best model using two features and the categorized cut-off values defined by C&R at 0.21 and 0.26 with respect to the Arch Index. Results show there is a statistically significant difference between the groups, as set by ANOVA (*F*_(2,211)_ = 10.18, *p* < 0.0001). Tukey’s HSD result shows a statistically significant difference in the mean value between the Low Arch and High Arch (0.000113 ± 0.000026 and 0.000135 ± .000026, respectively, *p* = .0001), as well as between the Normal (0.000126 ± 0.000024) and High Arch (*p* = 0.002). However, there were no differences between Normal and Low Arch (*p* = 0.191) for this two feature model. The Mean Bending Energy was the most important feature in this equation with a *p*-value < 0.001 compared to the Perimeter (*p* = 0.05). Including entropy improved the results and differentiated low arch from normal.

**FIGURE 4:**
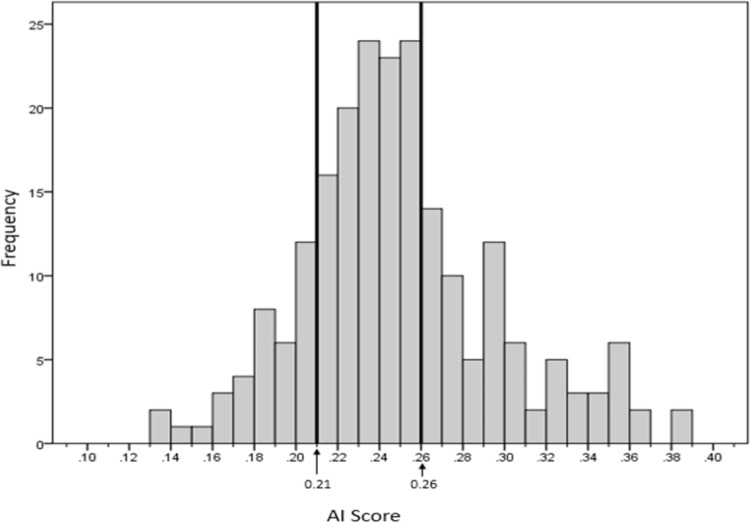
Distribution of arch indices determined with MBE + P using the Cavanagh and Rodgers proposed cut-off values.

**FIGURE 5:**
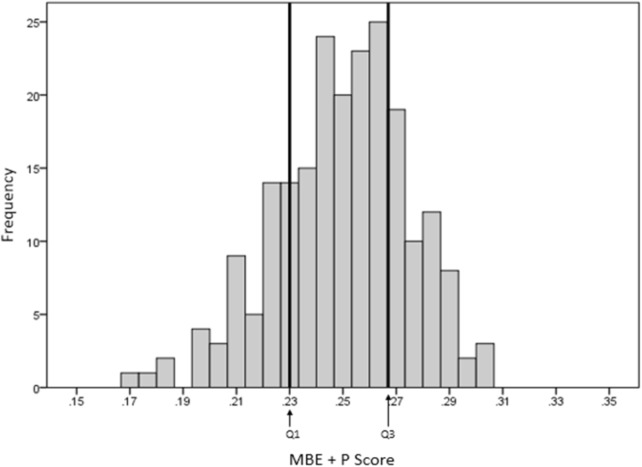
Distribution of scores using the MBE + P equation and cut-offs.

**Figures 4** and **5** show the respective AI distribution normalized to the C&R method scores and the MBE + P approach using quartiles to identify the low and high AI. The scores are normally distributed in both situations. The AI mean value for the C&R method was 0.25 and the standard deviation was 0.047. The mean value for the MBE + P approach was 0.25 and the standard deviation 0.025. The arrows indicate the first and the third quartiles of the distribution.

As clinicians typically divide feet into three groups, the distribution of MBE + P scores as shown in **Figure 5** were divided into three quartiles (Q1, Q2, and Q3). Q1 and Q3 are, respectively, showing high arch (low AI) and low arch (high AI).

The recommended cut-off for low and high AI differed slightly for the MBE + P to that of the C&R approach with high arch and low arch of 0.23 and 0.27 compared to the C&R of 0.21 and 0.26, respectively. The distribution of the footprints for each foot type for both methods from our sample is given in **Table 3**.

**Table 3:** Distribution of footprints for each category.

	High arch	Normal	Low arch
Cavanagh and rodgers	37	107	70
MBE + P	53	107	54

**FIGURE 6:**
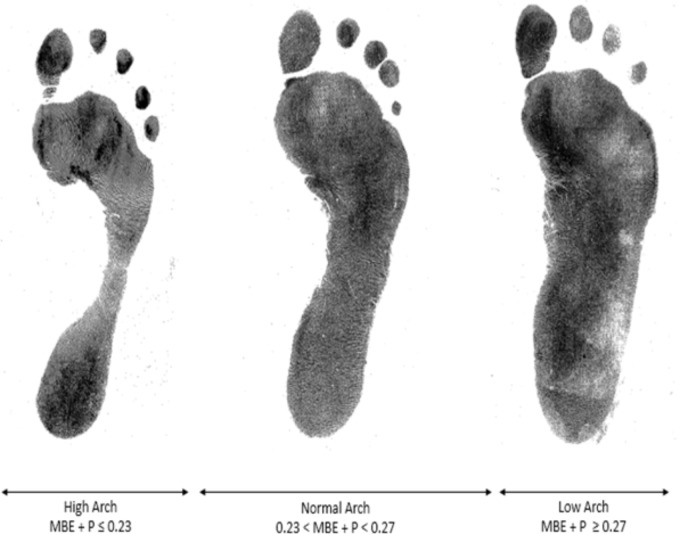
Visual MBE + P groups of arch height.

A representative example for each footprint category based on the cut-off scores given by the MBE + P equation are shown in **Figure 6**.

## Discussion

Automated analysis of foot type using MBE and perimeter is a novel approach, which may give clinicians a powerful, automated tool for timely identification of arch height type and possible risk of foot pathology. Previous methods using MRI, X-ray or CT images have shown good accuracy in Lin et al. (2015) but these methods are expensive, not widely available to clinicians, time consuming and expose participants to radiation. Combining bending energy with perimeter provided a different spatial analysis from the current Cavanagh and Rogers’ clinical approach. Our model distinguished the classes proposed by Cavanagh and Rogers providing a reliable tool in determining foot type. However, the mean energy combined with perimeter is more suitable for identification of high arch type, which is more difficult to assess clinically compared to low arch height (being flat footed). The better model based on the AICc in terms of accuracy was the MBE + SM + P model after removing collinearity from the complete model.

Mean Bending Energy plays an important role in the classification of a footprint due to the sensitivity of the measurement that highlights the middle longitudinal arch shape seen in the footprint. However, it depends on how accurately the footprint boundary/outline is presented. The more accurate and clearer the footprint boundary, the better the results. Comparing the two systems indicated that the MBE + P method was more sensitive in highlighting a high arch type as compared to the Cavanagh and Rodgers classification (**Figures 4**, **5**). The results of our approach lead to a greater number of footprints categorized as high arch compared to the C&R categorisation (**Table 3**). However, a review of these mismatched footprints by an experienced clinician indicated that our approach gave an average of 73% reliability for identifying any arch type, with up to 87% reliability in correctly classifying the high arch type. This indicated that the Cavanagh and Rodgers method may under-represent high arch. The C&R method relies on the total footprint area with respect to the area of the midfoot and can lead to some feet being classified as normal, due to the larger total area of the foot, but they may not be high arch foot type. Whereas the Mean Bending Energy + Perimeter relies on the global shape of the foot where the curvature associated with the arch height contributes the most weighting in determining MBE. Therefore, it is less sensitive to the shape of the foot and a better clinical tool that is automated and standardized.

Our automated analysis method utilizing mean bending energy, perimeter and entropy, identified specific frequency content in the footprints associated with specific directions in a localized region around each point of the perimeter of the footprint. This novel, simplified and robust approach provides clinicians a reliable method with faster results for assessment of arch height and a better understanding for predicting injuries associated with foot structure and posture.

## Author Contributions

JL, KK, JC, JJGL, and HJ contributed to this work. JL and JJGL wrote the scripts and conducted the MATLAB analysis. JC obtained ethical approval and collected the ink foot prints. JC and HJ undertook the statistical analysis and interpretation of the data. All authors contributed to writing main paper. KK gave technical support and conceptual advice. All authors discussed the results and implications and commented on the manuscript at all stages.

## Conflict of Interest Statement

The authors declare that the research was conducted in the absence of any commercial or financial relationships that could be construed as a potential conflict of interest. The reviewer CM declared a shared affiliation, with no collaboration, with one of the authors, JL, to the handling editor at the time of the review.

## Footnotes

1 http://ruckgaberbrueggemann.de/
